# Evaluation of two communication tools, slideshow and theater, to improve participants’ understanding of a clinical trial in the informed consent procedure on Pemba Island, Tanzania

**DOI:** 10.1371/journal.pntd.0009409

**Published:** 2021-05-14

**Authors:** Marta S. Palmeirim, Ulfat A. Mohammed, Amanda Ross, Shaali M. Ame, Said M Ali, Jennifer Keiser

**Affiliations:** 1 Swiss Tropical and Public Health Institute, Basel, Switzerland; 2 University of Basel, Basel, Switzerland; 3 Public Health Laboratory Ivo de Carneri, Chake Chake, Tanzania; Ministère de la Santé Publique et de la Lutte contre les Endémies, NIGER

## Abstract

**Background:**

Clinical trial participants are required to sign an informed consent form (ICF). However, information is lacking on the most effective methods to convey trial relevant information prior to inviting participants to sign the ICF, being particularly pertinent in low-income countries. A previous study on Pemba Island, Tanzania, found that a verbal information session (IS) was significantly better than providing an ICF alone. However, knowledge gaps remained. Building on these findings, we investigated the effect of adding a slideshow or a theater to the IS in the informed consent procedure of an anthelminthic clinical trial.

**Methodology/principal findings:**

A total of 604 caregivers were randomized into the control group that only received an ICF (n = 150) or an ICF plus one of three intervention strategies: (i) verbal IS (n = 135), (ii) verbal IS with a slideshow (n = 174) or (iii) verbal IS followed by a theater (n = 145). All modes of information covered the same key messages. Participants’ understanding was assessed using a semi-structured questionnaire. The mean score of caregivers in the control group (ICF only) was 4.41 (standard deviation = 1.47). Caregivers attending the IS alone were more knowledgeable than those in the control group (estimated difference in mean scores: 2.40, 95% confidence interval (CI) 1.95 to 2.86, p < 0.01). However, there was no evidence of an improvement compared to the IS only when participants attended a slideshow (0.09, 95% CI -0.53 to 0.35, p = 0.68) or a theater (0.28, 95% CI -0.27 to 0.82, p = 0.32). Three out of 10 key messages remained largely misunderstood, regardless of the mode of information group.

**Conclusions/significance:**

Our study confirmed that, in this setting, an ICF alone was not sufficient to convey clinical trial-related information. An IS was beneficial, however, additional theater and slideshows did not further improve understanding. Future research should explore methods to improve communication between study teams and participants for different key messages, study types and settings.

## Author summary

Legally, participants of clinical trials must sign an informed consent form prior to the start of a trial. To be truly informed, participants (or their caregivers, if underaged) need to understand the trial objectives, procedures, benefits, potential risks and their rights throughout the study. However, in regions where literacy and health literacy are still quite low, delivering information exclusively in written format using an informed consent form is not the best way to convey information. A previous study on Pemba Island, Tanzania, found that a spoken information session in addition to the informed consent form alone, significantly improved the clinical trial-related knowledge of the caregivers of participants. However, not all questions could be answered correctly. In the current study, we explored the addition of two further modes of information to the information session: a slideshow or a theater show. Caregivers’ understanding was measured using a questionnaire. We confirmed that having an oral information session is significantly better than only receiving the informed consent form. However, adding a slideshow and theater did not further improve caregivers’ knowledge. We conclude that future studies should work on improving these modes of information or explore new ones.

## Introduction

Obtaining informed consent from participants is an ethical obligation in clinical research that has to include five core elements: voluntarism, capability, disclosure, understanding and decision. According to del Carmen, the most challenging element to achieve is understanding [1]. Too often researchers rely on the informed consent form (ICF) alone to present clinical-trial related information to participants [2]. While legal, this does not ensure the participant’s true understanding and, therefore, does not constitute a true informed consent. This issue is particularly problematic in settings where health literacy levels are still quite low. A literature review from 2014 found that, in African settings, a large proportion of trial participants had difficulties grasping concepts such as randomization, placebo and the difference between participating in clinical research and seeking medical care [3]. This inability to distinguish research from medical care is also known as “therapeutic misconception” and it is related to low literacy and educational levels, heavy burden of disease and “the overriding impact of illness, suffering and poverty on decision-making” [3]. In such conditions, people are particularly susceptible to manipulation, and are more likely to agree to research for the same reasons they agree to treatment [4,5].

It is the investigators responsibility to ensure an appropriate level of understanding prior to having a potential participant sign an ICF [4]. Three important steps are required to ensure a truly informed decision. The first step is to define which concepts and study procedures need to be understood by participants before they consent. The second step is to find effective methods to communicate these to participants, taking into account cultural values and constraints. The final step is to measure people’s understanding of the identified concepts and study procedures using, for example, a questionnaire. Currently, there are no validated and standardized tools to do so [3], and an official guidance through, for example, the Good Clinical Practice guidelines, is not available.

In our previous study of the effectiveness of modes of communication for informed consent for an anthelminthic trial on Pemba Island, we developed a pamphlet and conducted an oral information session (IS), and compared their effect, separately and combined, on the caregivers’ knowledge about the clinical trial [6]. We found pamphlets were not an effective method to communicate information but that a verbal IS resulted in higher scores in the questionnaires testing knowledge. However, there were still some key messages that were not well understood by caregivers. Thus, new communication methods needed to be explored. Building on this previous study, in the current study we compared the effects of a verbal IS alone or in combination with a slideshow or a theater.

## Methods

### Ethics statement

The current study was embedded in a school-based, open-label, randomized clinical trial comparing the efficacy, safety and acceptability of a new chewable tablet formulation of mebendazole to the standard solid tablet formulation against soil-transmitted helminth infections. The trial included children aged 3 to 12 years old and took place in the primary schools of Bwagamoyo, Furaha, Piki and Wawi on Pemba Island, Tanzania, from July to October 2019. The methodology and results of this clinical trial (number NCT03995680, ClinicalTrials.gov) have been published elsewhere [6]. Caregivers, i.e. parents or guardians of the children, were asked if they would like to participate in the current study. Written informed consent was obtained from all of those who agreed. Those who could not read provided a thumbprint and the ICF was signed by an impartial witness ensuring all important information was conveyed correctly.

### Sample size

We used the formula for comparing two proportions of Kirkwood and Sterne to establish the sample size [7]. Caregivers’ knowledge about the clinical trial was measured using a questionnaire. Assuming that the proportion of caregivers answering a question correctly in the IS would be 25% and with an additional intervention 40%, 150 participants per arm would be required to detect this difference as significant with 80% power.

### Methods for conveying information

All children who were potentially eligible for the clinical trial received an ICF in Swahili with information concerning the clinical trial to hand to their caregivers. Children were also asked to tell their caregivers on which day they were invited to join an IS. Upon arrival to the school where the IS took place, caregivers were assigned to a control group or one of three interventions: (i) verbal IS alone, (ii) verbal IS followed by a theater, and (iii) verbal IS with a slideshow. The group of caregivers who served as the controls responded to the questionnaire before receiving any information (iv).

The first intervention consisted of an oral presentation of all key messages included in the ICF (S1 Text). In the second intervention, a power point slideshow with pictures and key words was projected on the classroom wall during the oral presentation (see S2 Text). The third intervention included the verbal IS (as in the first intervention group), followed by a theater show performed by students from the participating secondary schools. In preparation for the intervention, the students received some training on acting. An outline of the script for the theater was drafted by our research team but allowed the actors to make adaptations as long as all key messages were included (S3 Text). To avoid bias, the same speaker conducted all ISs and the content was standardized. In every intervention group, caregivers were encouraged to ask questions before deciding whether their child should participate in the trial or not.

### Group allocation and study procedures

**Fig 1:**
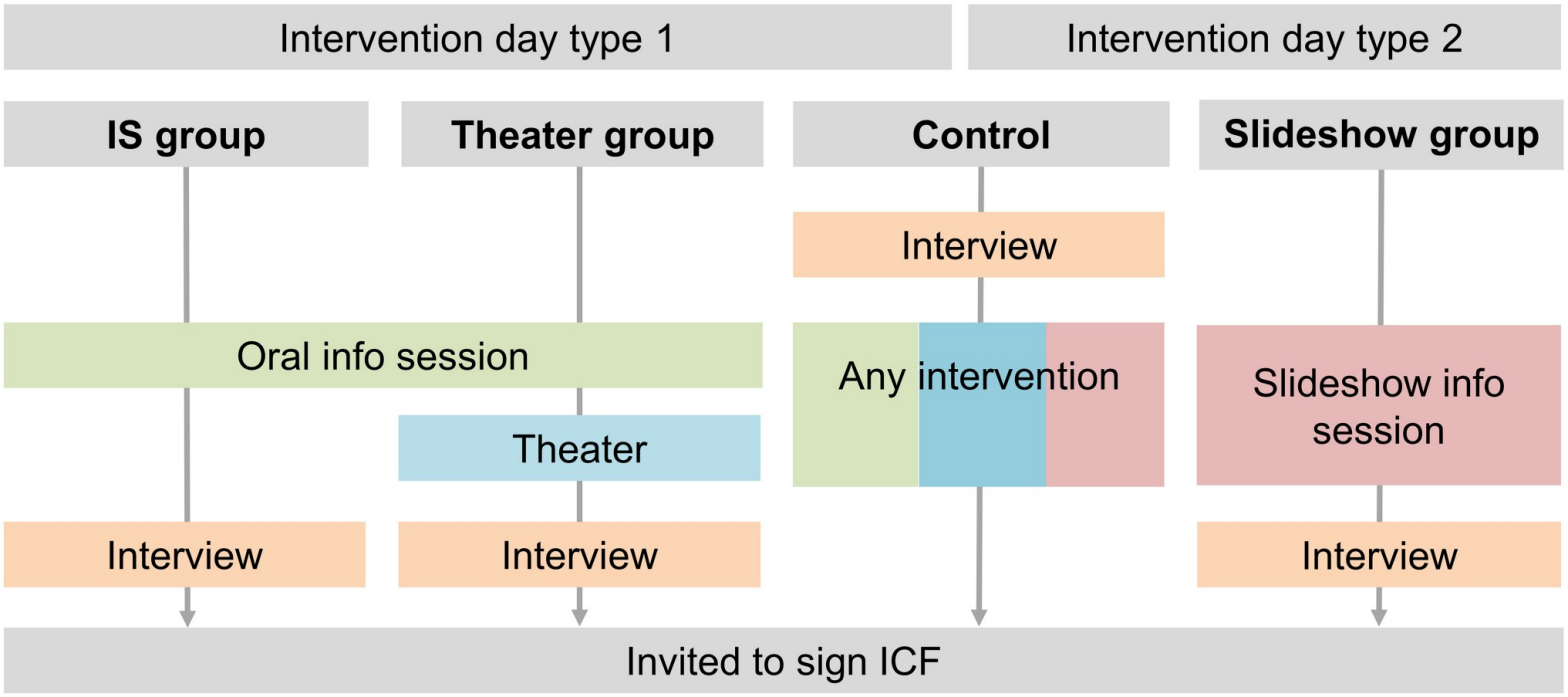
Flow chart describing the procedures during the two types of intervention days. ICF = informed consent form, IS = information session.

All interventions were implemented in each school. On each day, the IS took place however the theater and slide shows took place on alternate days for practical reasons (Fig 1). On the theater days, there were two intervention groups (IS only, and IS plus theater) and on slide show days, only one (IS plus slideshow). Caregivers were randomly allocated to one of the groups as soon as they arrived to the classroom where the IS took place. Depending on the type of IS day, two (control and slideshow) or three (control, verbal IS alone and verbal IS plus theater) colored and labelled cards were shuffled in an opaque bag and chosen at random to give to each caregiver as they entered the classroom. Interviewers started by interviewing all caregivers with a card labelled as control. Once these caregivers had all been interviewed the intervention took place. Interviews took place either in the IS room or in an adjacent room, when the IS room did not allow sufficient privacy. After following one of the three interventions all caregivers from the control group were invited to sign an ICF. The caregivers belonging to the intervention groups were interviewed and then invited to sign an ICF. No caregiver was invited to sign an ICF without receiving all important information concerning the clinical trial.

### Key messages for participants

**Fig 2:**
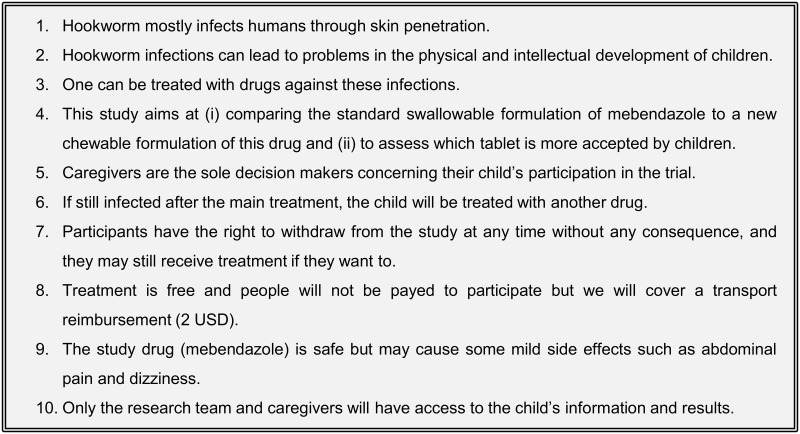
Key messages conveyed during all three types of IS.

The ISs included 10 key messages we considered participant caregivers should understand prior to signing the ICF (Fig 2). Apart from the objectives of the clinical trial (#4), all key messages were the same as those in our previous study [6].

### Questionnaire and data collection

The questionnaire consisted of three parts: a set of 10 main questions (part A), followed by one larger question related to the study procedures (part B), and three true/false statements (part C).

Part A: all spontaneous answers were recorded (A1) and, if the participant did not have a spontaneous answer to one of these questions, the interviewer moved to a multiple choice version with four options of the same question (A2). If the participant was unable to choose one of the options, the interviewer marked the answer as “does not know”. After the first question–“what is hookworm and how does it infect us?”–regardless of the interviewees response we told them that “hookworm is a type of worm that could infect us when we walked barefoot, for example”. This way, all caregivers were minimally informed to be able to respond to the following questions.

Part B: Caregivers were then asked “what will your child have to do if he/she participates in this clinical trial?”. All spontaneous responses to this question were recorded by the interviewer (B1) and, if the caregiver did not mention some of the seven key messages we expected him/her to mention, interviewers presented statements related to those key messages and asked the caregiver to decide whether this statement was true or false (B2).

Part C: Finally, a set of three true/false statements aimed at assessing whether caregivers understood we were conducting research and not providing treatment to all children or community members.

The full questionnaire is provided in S1 and S2 Tables. Answers were recorded using a semi-structured questionnaire on tablet computers. Each interview lasted about 15 minutes. The questionnaire was orally administered in Swahili by eight interviewers who underwent three days of training. Interviewers recorded the interviewee’s age, gender and whether they could read or not. Each caregiver was only interviewed once, regardless of how many children they had participating in the clinical trial. Whereas caregivers assigned to one of the three interventions were interviewed immediately after the intervention, caregivers in the control group responded to the questionnaire before being exposed to any mode of information.

Each child participating in the clinical trial responded to a short survey inquiring about possession of specific household assets (soap, radio, television, computer, cell phone, refrigerator, fan, bicycle, scooter, car, tractor and electricity) to use as a proxy for socioeconomic status.

### Scoring the questionnaire

Spontaneous answers were categorized as correct or incorrect based on whether or not the response had been mentioned during the intervention. For simplicity, some responses that were correct or partially correct but not mentioned in the IS, were not classified as correct because we aimed at measuring the gain in knowledge resulting from each type of intervention, rather than the caregivers’ background knowledge. All questions but two had a single correct answer. For all probed answers, whether multiple choice or true/false, there was only one correct answer. No overall score was produced for the probed answers.

### Statistical analysis

An overall score of the spontaneous answers was calculated by summing the number of correct answers to the ten main questions (only the answers to the main questions were considered (not the sub-questions) and, for questions with more than one correct answer, a caregiver got a point if he/she mentioned at least one).

The effect of method of communication on the proportion of caregivers correctly answering each question spontaneously was estimated using logistic regression, and on the overall score of answers using linear regression. In both models, a random effect was included to account for clustering by day. The estimates were adjusted for caregiver’s age, interviewer, socioeconomic group, sex, school and whether the caregiver could read or not. The socioeconomic index was calculated by summing the number of assets owned by a child’s household and divided into five categories. The caregivers’ age was split into three categories: ≤25, 26 to 50, and ≥ 51 years.

We did not perform any analysis for answers to probed questions, spontaneous answers to the question “what will your child have to do if he/she participates in the trial?”, and true/false statements since the sample size in these groups was too few.

## Results

The study took place in four schools and included a total of 604 participants. A total of 17 sessions took place. There were 150 participants allocated to the control group, 135 to the verbal IS alone group, 174 to the slideshow IS group, and 145 to the verbal IS plus theater group. Interviewees had an average age of 38 years, 62% were women and 64% stated they could read; these were balanced across the different groups (Table 1).

**Table 1:** Characteristics of interviewees stratified by group.

	Control (n = 150)	IS (n = 135)	IS + slideshow (n = 174)	IS + theater (n = 145)
Women [n (%)]	62 (41)	57 (42)	60 (35)	53 (37)
Age [years (SD)]	41 (14)	40 (13)	39 (13)	41 (12)
Literate [n (%)]	88 (59)	88 (65)	111 (64)	99 (68)

### Knowledge of the key messages about helminths

Participants who attended the verbal IS gave overall more correct spontaneous answers to the ten main questions than those in the control group (Table 2). For six of the ten main open questions (questions 1, 2, 7, 8, 9, 10) and four answers to the sub-questions (questions 4, 7 and 9) there was a significant difference between the proportions of respondents giving correct answers among the groups. Two questions were answered correctly by participants in all of the groups, while three questions resulted in few correct spontaneous answers overall. For example, regardless of the mode of information, most caregivers knew that one can be treated against hookworm and that caregivers are the ones who decide whether their child should participate in a clinical trial or not. On the other hand, most caregivers did not understand that they can withdraw from the study without any consequences.

**Table 2:** Number of caregivers spontaneously answering each question correctly (Part A1). IS = information session. Data are n (%, using the total number of caregivers in each group as a denominator).

	Caregiver group				P-value for the effect of IS compared to control	P-value for the effect of slideshow compared to info session	P-value for the effect of theater compared to info session
	Control (n = 150)	IS (n = 135)	IS + slideshow (n = 174)	IS + theater (n = 145)			
1. **How does hookworm infect us**?							
Through skin penetration, when we walk barefoot	27 (18)	117 (87)	146 (84)	130 (90)	<0.01	0.98	0.52
2. **Why is it bad for your child**?							
My child might not grow well	49 (33)	91 (67)	97 (56)	96 (66)	<0.01	0.17	0.90
My child might not do well at school	4 (3)	24 (18)	48 (28)	33 (23)	<0.01	0.99	0.52
3. **Can one be treated**?							
Yes, with a drug	139 (93)	130 (96)	162 (93)	140 (97)	0.33	0.78	0.79
4. **Why are we doing this study**?							
To assess which tablet kills more worms	3 (2)	25 (19)	45 (26)	30 (21)	<0.01	0.05	0.28
To assess which tablet children like more	2 (1)	5 (4)	3 (2)	11 (8)	0.12	-	0.36
5. **Who decides if your child participates**?							
Caregiver(s)	147 (98)	134 (99)	172 (99)	143 (99)	0.61	-	-
6. **What if your child still has hookworm**?							
Will be re-treated	116 (77)	124 (92)	153 (88)	136 (94)	0.11	0.70	0.14
7. **Can you give up participating**?							
Yes	19 (13)	37 (27)	32 (18)	33 (23)	0.01	0.39	0.64
*And what happens if he/she gives up*?							
There are no consequences	7 (5)	9 (7)	13 (8)	11 (8)	0.81	0.60	0.97
8. **What about payment**?							
You will receive transport reimbursement	93 (62)	126 (93)	159 (91)	131 (90)	<0.01	0.26	0.86
9. **Is mebendazole safe**?							
Yes	89 (59)	120 (89)	157 (90)	130 (90)	<0.01	0.81	0.25
*He/she might have some side effects or nothing at all*?							
Might have some side effects	40 (27)	67 (50)	89 (51)	74 (51)	<0.01	0.66	0.46
*What are potential side effects*?							
Belly ache	15 (37)	32 (48)	38 (43)	35 (47)	0.10	0.32	0.57
Dizziness	17 (43)	46 (69)	74 (83)	56 (76)	0.40	<0.01	0.09
10. **Who can see your child’s information**?							
Caregivers and investigators	21 (14)	32 (24)	45 (26)	52 (36)	<0.01	0.22	0.63

**Note:** Main questions are highlighted in bold and sub-questions in italic. The p-values were calculated using logistic regression with a random effect for school, and adjusted for caregiver’s age, interviewer, socioeconomic group, day, sex and whether the caregiver could read or not. Some questions had two correct answers that were analyzed separately.

**Fig 3:**
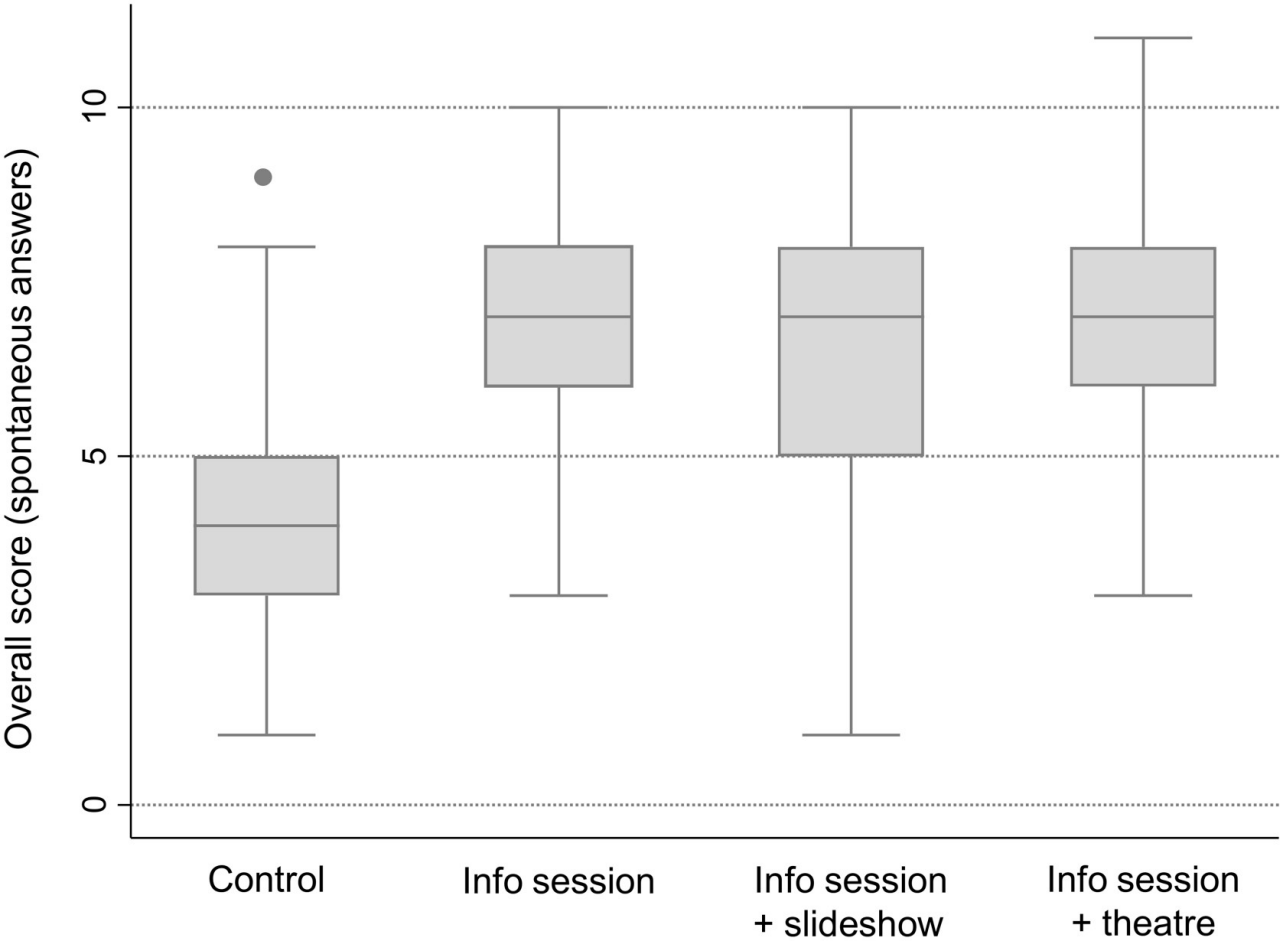
Boxplot of the overall score of spontaneous answers to the ten open-ended questions by caregiver group. Lines in a box represent the first quartile (upper line), median (middle line), third quartile (lower line); whiskers represent the maximum and minimum, and outliers are plotted as individual points.

When analyzing the overall score of the spontaneous answers to these ten open questions, the mean score of caregivers in the control group was 4.41 (standard deviation (SD) = 1.47) compared to a higher score of 6.83 (SD = 1.62) in the IS group (estimated difference in score: 2.40, 95% confidence interval (CI) 1.95 to 2.86, p < 0.01) (Fig 3). However, there was no evidence that having been exposed to a slideshow or a theater, in addition to the IS, had a further benefit on the overall scores (-0.09, 95% CI -0.53 to 0.35, p = 0.68 and 0.28, 95% CI -0.27 to 0.82, p = 0.32, respectively).

If a caregiver could not respond spontaneously to one of the ten main questions, the interviewer probed for an answer by presenting the caregiver with four options where one was correct (multiple choice format; as described for part A2) (S1 Table). We found that, overall, when probed, caregivers were less likely to respond correctly to almost all questions, when compared those answering spontaneously. As for the spontaneous answers, correct responses tended to be higher in the three intervention groups, compared to the control group, with a few exceptions (Table 3).

**Table 3:** Caregivers choosing the right answer to the ten main questions when probed (Part A2). Data are n (%).

	Control	IS	IS + slideshow	Verbal IS + theater
How does hookworm infect us?	17/63 (27)	7/10 (70)	11/21 (52)	4/8 (50)
Why is it bad for your child?	6/31 (19)	0/8 (0)	17/26 (65)	5/13 (38)
Can one be treated?	1/3 (33)	NA	1/3 (33)	1/1 (100)
Why are we doing this study?	23/126 (18)	18/47 (38)	17/62 (27)	20/45 (44)
Who decides if your child participates?	0/1 (0)	NA	1/1 (100)	NA
What if your child still has hookworm?	15/23 (65)	3/3 (100)	16/17 (94)	3/3 (100)
Can you give up participating?	0/15 (0)	0/7 (0)	0/4 (0)	0/3 (0)
What about payment?	15/40 (38)	5/5 (100)	10/11 (91)	2/2 (100)
Is mebendazole safe?	3/58 (5)	1/13 (8)	2/17 (12)	0/10 (0)
Who can see your child’s information?	1/7 (14)	1/1 (100)	0/1 (0)	NA

**Note:** We removed interviewees who answered spontaneously. IS = information session. NA = not available because all caregivers responded spontaneously.

### Knowledge of study procedures

After having completed the ten main questions, caregivers where asked “what will your child have to do if he/she participates in this clinical trial?”. All spontaneous answers were recorded (part B1) and, if caregivers did not mention all the procedures we expected them to, a true/false statement was presented to them concerning each of the procedures they did not mention (part B2).

Caregivers’ spontaneous answers tended to be most often correct when caregivers underwent one of the three interventions (Table 4). The most frequently recalled key message was that children will need to provide stool samples. However, most participants did not know how many stool samples. The few people who did know the correct number of stool samples all belonged to either the slideshow or the theater group. All caregivers in the three intervention groups who mentioned the blood sample knew it would be a small sample. Caregivers in these groups were also more likely to remember that girls would undergo a pregnancy test. Other messages such as “telling us how he/she feels after treatment” and “allowing a nurse/doctor to examine him/her” were barely mentioned by caregivers, regardless of their group. However, when presented with true/false statements about these issues, the vast majority answered correctly, even in the control group. The detailed numbers of caregivers mentioning each of the key messages spontaneously (with sub-questions) or responding correctly to the true/false statements concerning each key message not mentioned spontaneously are presented in S1 Table.

**Table 4:** Caregivers mentioning each of the key messages or responding correctly to the true/false statements concerning each key message not mentioned spontaneously (Part B1 on the left and B2 on the right). IS = information session. Data are n (%).

Spontaneous answers	Control (n = 150)	IS (n = 135)	IS + slideshow (n = 174)	IS + theater (n = 145)	True/false statements	Control	IS	IS + slideshow	IS + theater
**What will your child have to do if he/she participates in this clinical trial?**									
Give us stool samples	76 (51)	119 (88)	136 (78)	134 (92)	Give us one stool sample	2/74 (3)	1/16 (6)	1/38 (3)	0/11 (0)
How many? (four)	0 (0)	0 (0)	11 (8)	9 (7)					
Tell us how he/she feels after treatment	1 (1)	1 (1)	5 (3)	1 (1)	Tell us how he/she feels after treatment	109/149 (73)	121/134 (90)	154/169 (89)	133/144 (92)
Provide a blood sample	7 (5)	25 (19)	42 (24)	38 (26)	Provide a small blood sample	107/143 (75)	108/110 (98)	127/132 (96)	107/107 (100)
Big or small? (small)	6 (86)	25 (100)	42 (100)	38 (100)					
Allow a doctor/nurse to examine him/her	5 (3)	9 (7)	25 (14)	12 (8)	Allow a doctor/nurse to examine him/her	129/145 (89)	124/126 (98)	148/149 (99)	132/133 (99)
Pay for treatment	0 (0)	0 (0)	1 (1)	0 (0)	Pay for the treatment	88/150 (59)	101/135 (75)	140/174 (81)	107/145 (74%)
Drink water during treatment day	0 (0)	0 (0)	1 (1)	0 (0)	Not drink water during treatment day	83/150 (56)	60/135 (44)	96/174 (56)	78/145 (54)
Make a pregnancy test, if a girl	5 (3)	15 (11)	25 (14)	25 (17)	Provide a urine sample for a pregnancy, if she is 9 years old	17/145 (12)	7/120 (6)	7/149 (5)	11/120 (9)
Starting at what age? (10 years)	2 (40)	10 (67)	18 (72)	18 (72)					
**Why are we (research team) here?**									
					To treat all the people in the village.	85/150 (57)	104/135 (77)	130/174 (75)	117/145 (81)
					To do research and treat only children who are in our study.	100/150 (67)	91/135 (67)	133/174 (76)	107/145 (74)
					To treat all the children in the school.	83/150 (55)	106/135 (79)	126/174 (72)	113/145 (78)

### Misconceptions about the therapy

Finally, three true/false statements aiming at assessing whether caregivers understood the difference between research and medical care were presented to all caregivers (questionnaire part C). We found that 67–74% of caregivers understood that our study would only treat study participants, answering that the statement “we are here to do research and treat only children who are in our study” was true. However, there was also a considerable portion of caregivers answering that the statements “we are here to treat all the people in the village” (19–25%) and “we are here to treat all the children in the school” (21–28%) were true, suggesting they did not fully grasp that we were doing research and not providing medical care.

Answers to all questions of the questionnaire stratified by communication method and spontaneous and probed answers are presented in S2 Table.

## Discussion

Building on a previous study that also took place on Pemba Island, Tanzania [6], we tested the effect of a slideshow and a theater in addition to the verbal IS on caregivers’ knowledge concerning the clinical trial. We confirmed that providing caregivers with an IS significantly improved their knowledge, when compared to the control group that only received an ICF. However, we did not find any evidence of an additional benefit from complementing the IS with a theater or slideshow.

The result that an IS is pivotal for increasing participant’s knowledge is in line with our previous study, although the two studies are not entirely comparable since in the previous study the questionnaire consisted of closed-ended questions and in the current study almost all questions were open-ended. Closed-ended questions, which might overestimate levels of understanding [8], were a limitation of our previous study. Perhaps because closed-ended questions may be more of a measure of recognition and recall [8], in this study, we found that interviewees in the control group of the previous study were more likely to respond correctly to some questions compared to those in the control group of the current study with open-ended questions, since this approach allows measuring more complex features of understanding [8].

Some key messages were easily captured by caregivers, but others continue to be widely misunderstood. One example of good understanding was the mode of transmission of hookworm. In contrast, caregivers scored quite low regarding the aim of the study (many responded we aimed at treating children), the right to withdrawal (most caregivers believed the child would not receive treatment if he/she withdrew) and confidentiality of data (about half of the caregivers responded that only caregivers would be able to see their child’s data), which are all very important issues. Similar results have been reported elsewhere [3]. Although caregivers in the slideshow and theater groups were more likely to know the number of stool samples their child would need to provide and the potential consequences of hookworm infections on children, we believe that the gains of these interventions on isolated questions were not sufficient to justify the extra effort they require. Slideshows require a projector that needs to be connected to electricity and often schools do not have electricity. The theater requires finding actors, providing training and a considerable amount of rehearsing.

Our results point out the need to, first, explore potential reasons behind the lack of understanding of some key information and, second, to investigate new methods of conveying clinical trial-related information.

Concerning the first point, there might be several explanations as to why people do not always understand certain topics. Besides the communication method not being the most adequate, it could be that the messages we try to convey are too complex, particularly in contexts with low educational levels, or that people are not aware of the importance of understanding and, therefore, do not make the effort that is required to understand. A study in Romania analyzing the behavior of 68 patients during the informed consent process found that some showed little interest in understanding; receiving treatment and feeling better seemed to be their main concern [4]. This could be linked to the fact that, in some contexts, people do not feel responsible for making this decision for themselves. As highlighted in our previous study, perhaps participants trust that, if the government, the research ethics committees and the schools have approved this study, then they can trust us and, hence, do not need to fully understand the study before enrolling their children [5]. Molyneux and colleagues conducted a study on the relationship between trust and informed consent among community members on the Kenyan Coast and suggest that patients often trust that the clinician/researcher will always use his/her knowledge for the good of the patient, choosing to allow them to function as a substitute decision maker [4,5]; hence the lack of interest to understand. Focus group discussions and in-depth individual interviewers should take place to help reveal the factors leading to this problem on Pemba Island, Tanzania.

Once we identify the reasons behind people’s lack of understanding, novel interventions should be explored. For example, a video could be presented, which has been found to increase clinical trial-related knowledge in previous studies [9–11].

Our study had some important limitations. The first is that interviewers were not blinded to the group caregivers were assigned to, which could lead to some bias in the way they performed the interview. Second, we cannot exclude that caregivers did not read the ICF they were given by their children, and this is particularly relevant for the control group. The only way of guaranteeing everyone in the control group has been exposed to the information on the ICF would be to read it out loud with caregivers before interviewing them. However, this would have changed the meaning of the control group since, in real life, the ICF is often just handed to people and there is usually no guarantee they have read it or had someone read it to them.

In conclusion, our findings reveal that attending a verbal IS significantly increases the knowledge of participants or their caregivers, when compared to providing an ICF alone. Complementing the verbal IS with a slideshow or a theater did not represent any clear advantage in this study. Several key messages persisted as difficult for caregivers to understand. Therefore, future studies investigating why this is the case should be implemented in order to create sufficient evidence to design ISs adapted to different types of studies and settings.

## Supporting information

S1 Text Oral information session speech. (DOCX)

S2 Text Slideshow projected and presented to caregivers in parallel to the information shared orally. (PDF)

S3 Text Script for guiding the theater presented to caregivers after an oral information session. (DOCX)

S1 Table Number (%) of caregiver responses to each question stratified by spontaneous or probed answers and by caregiver group. (DOCX)

S2 Table Number (%) of caregivers mentioning each of the key messages spontaneously or responding correctly to the true/false statements concerning each key message not mentioned spontaneously by caregiver group. (DOCX)
