# Supplementary material for: Evaluation of two communication tools, slideshow and theater, to improve participants’ understanding of a clinical trial in the informed consent procedure on Pemba Island, Tanzania
Source: PLoS Negl Trop Dis. 2021 May 14;15(5):e0009409. doi: 10.1371/journal.pntd.0009409 (PMC8153490; doi:10.1371/journal.pntd.0009409)
Supplement: S1 Text — (DOCX) [file pntd.0009409.s001.docx]

**S1 Text.** Oral information session speech.

During every oral information session, the local lead staff member presented the information below to caregivers. Very simple language was used to reach people of all literacy levels. The information session was done in the local language, Swahili.

- We come from the Public Health Laboratory in Chake Chake and we doing a research study about belly worms.
- We are not here to treat all the children or people in the village, only the children who are included in our study.
- We will explain the whole research project and you can ask all the questions you want to.
- Have you ever heard of belly worms that can infect us and our children?
- Some worms infect us when we drink dirty water or swim in a river, for example. However, that is not the case of the type of belly worm we are going to talk to you about today.
- Our research is about one of these worms and it is called hookworm (*write hookworm on the chalk board*).
- Hookworm is a worm that is in the soil and, if a person walks without shoes and steps on it, the worm can go into the foot through the skin. Then the worm goes through the blood all the way to the belly where it will live.
- Many children in Pemba have these worms in their belly. Maybe even your child…
- These worms can cause many problems. For example:
- Your child may not grow well
- Your child may not be able to concentrate in school
- When he/she grows up, he/she might not be able to work so well
- There are different medicines that kill this hookworm that can live in children’s bellies.
- In our research, we want to test one medicine that kills this worm. This medicine is called mebendazole (*write it on the board and show the mebendazole tablet*).
- Mebendazole is a drug that has been used for almost 50 years so we know it is safe. Sometimes it can cause some side effects like a bellyache or dizziness but it does not cause anything dangerous.
- Your child has probably received mebendazole before, at school or at the clinic.
- Maybe you have noticed that these tablets are very big which is difficult to give to small children. Also, they don’t taste very good when you chew them. Mebendazole was never made to be chewed – it was made to be swallowed whole with water and if you chew maybe it does not work so well.
- Because it is almost impossible for a small child to swallow it whole, the company invented a new mebendazole tablet you can chew and it still works well.
- So in our study we have to main objectives:
- We will check if this new tablet to chew kills more hookworm in your child’s belly than the old one to swallow.
- We will check which tablet children seem to like better.
- For this research project, we will include 400 children aged between 3 to 12 years old.
- You will decide yourself if you want your child to participate. It is not us, or the doctors, or the teachers, or your neighbor who decide – it is the parents of the child.
- If you decide your child should participate in this study and receive treatment he will have to do several things:
- He/she will have to give us 2 stool samples so we can find out if he/she has hookworm in his belly or not.
- He/she will have to let a doctor and a nurse check his health.
- He/she will have to give us a small sample of finger blood to check if he/she has anemia.
- He/she will receive the medicine called mebendazole (either the old one or the new one – this is decided by chance). The treatment is one day only. We will bring tea and food for your child. It is very important that your child comes for the treatment day, we will tell you in advance when it is going to happen.
- If your child is a girl and over 10 years old, we will ask her for a urine sample just to confirm that she is not pregnant.
- The day after receiving the treatment, your child has to come to school so we can check how he/she is feeling.
- Two or three weeks after he/she is treated we will ask him/her for 2 more stool samples to check if the treatment killed the worms or if he/she still has hookworm in his belly.
- These are the things we will ask from your child. Any questions?
- If you want your child to participate in our research and receive the treatment, we will ask you to sign the paper you have with you. But not quite yet. We can help you sign it on your way out.
- If now you decide you want your child to participate but then you change your mind that is no problem at all. You can give up at any moment without any consequences. Your child can still receive treatment if you want.
- It could happen that the mebendazole does not kill the hookworm in your child’s belly. If this happens, we will give him another treatment, albendazole and ivermectin.
- It is important to mention this treatment is for free – you do not have to pay anything. We do not pay you if your child participates but we will give you 3,000 shillings because you came today to the meeting to pay for your transport to the school, even if you decide you do not want your child to be part of the study.
- Also, the research team members are the only people who can see your child’s personal information and results. We will not show it to the teachers, we will not show it to your neighbors, nobody. Only you and us will see.
- Our phone numbers are on the papers that you will keep in case you ever have any questions, even after leaving today. Any questions now?
