# Supplementary material for: Evaluation of two communication tools, slideshow and theater, to improve participants’ understanding of a clinical trial in the informed consent procedure on Pemba Island, Tanzania
Source: PLoS Negl Trop Dis. 2021 May 14;15(5):e0009409. doi: 10.1371/journal.pntd.0009409 (PMC8153490; doi:10.1371/journal.pntd.0009409)
Supplement: S2 Text — (PDF) [file pntd.0009409.s002.pdf]

S2 Text. Slideshow projected and presented to caregivers in parallel to the information shared orally (presented in S1 Text). * Pictures that cannot be shown due to copy-right issues.

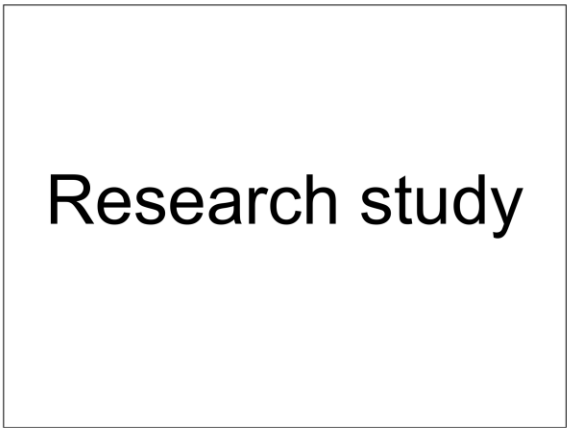

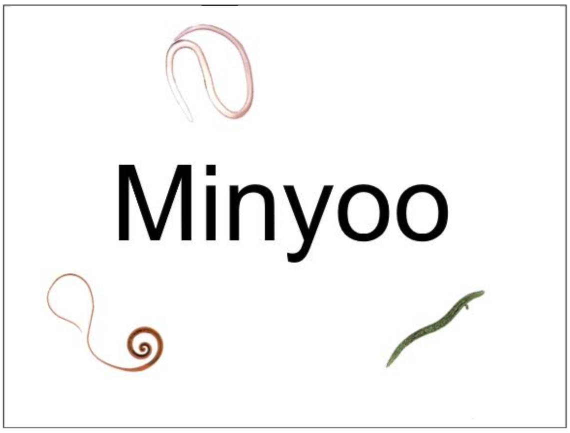

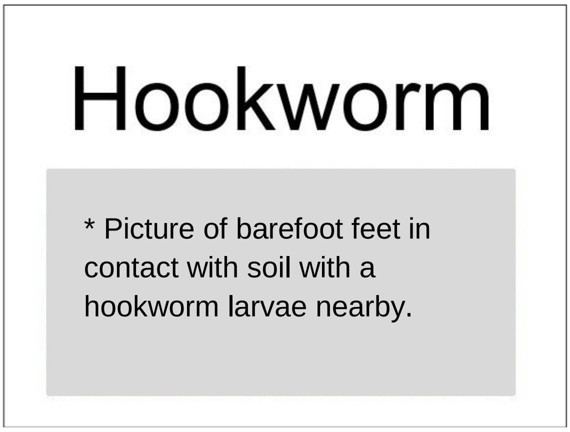

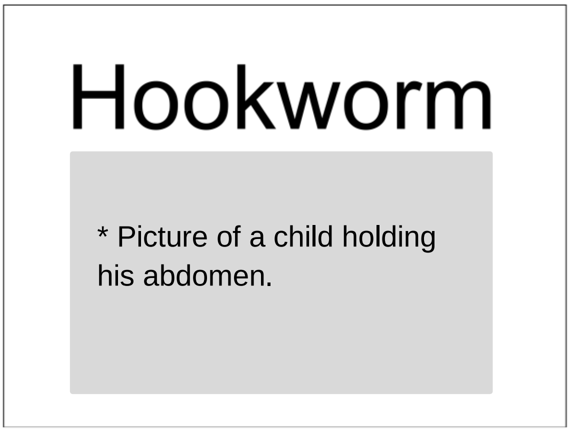

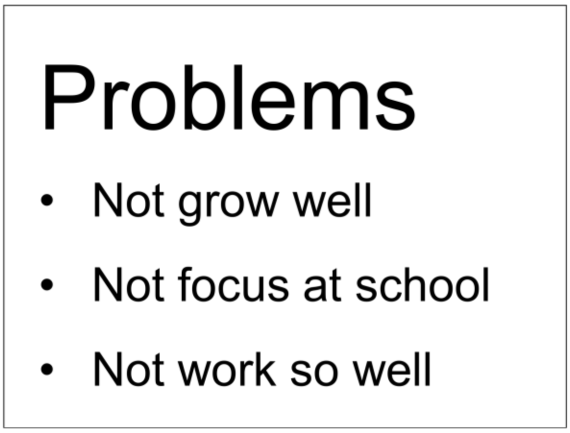

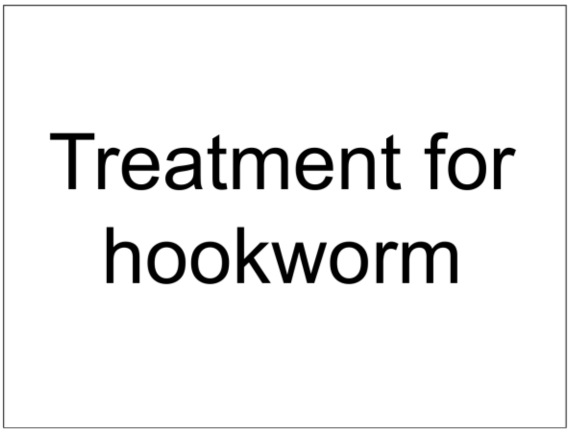

MEBENDAZOLE

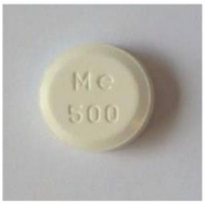

- 50 years
- safe
- dizzy or belly ache

MEBENDAZOLE

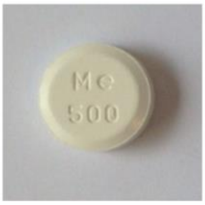

- too big
- bad taste

New MEBENDAZOLE

* Person chewing a tablet

Chew before swallowing

MEBENDAZOLE

Old swallow

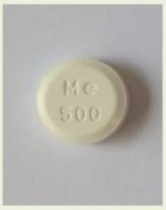

New chew

* Person chewing a tablet

2 questions

1. Which one kills more minyoo?

Old swallow

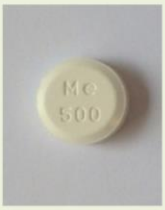

New chew

* Person chewing a tablet

2. Which one do children like more?

Old swallow

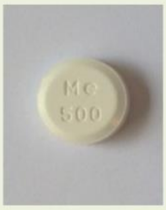

New chew

* Person chewing a tablet

3 to 12 years old

Check stool

400 with hookworm

200

200

200

Old swallow

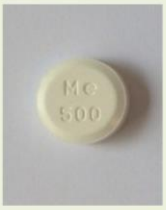

200

New chew

* Person chewing a tablet

PARENT decides

What child needs to do?

2 stool samples

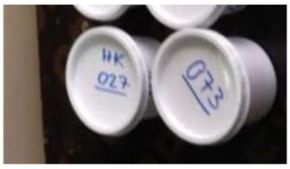

Doctor check

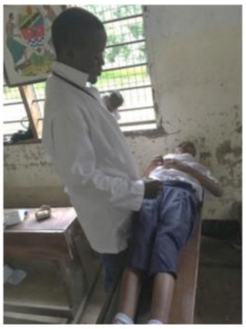

Small blood sample

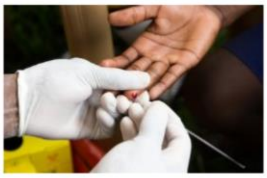

Girl and **10** years or older

Pregnancy test

Eat mebendazole

Tell us how feels day after

Wait 2 or 3 weeks

2 stool samples again

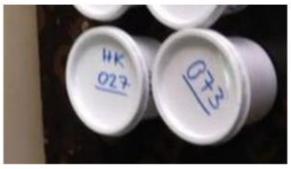

No problem to change your mind

Albendazole and ivermectin

Treatment is FREE

3’000 TSh transport

Only you and us see results

Thank you

Questions?
