# Supplementary material for: Evaluation of two communication tools, slideshow and theater, to improve participants’ understanding of a clinical trial in the informed consent procedure on Pemba Island, Tanzania
Source: PLoS Negl Trop Dis. 2021 May 14;15(5):e0009409. doi: 10.1371/journal.pntd.0009409 (PMC8153490; doi:10.1371/journal.pntd.0009409)
Supplement: S3 Text — (DOCX) [file pntd.0009409.s003.docx]

**S3 Text.** Script used as a base for the theater presented to caregivers after a spoken information session. The secondary school students who performed the theater were given the freedom to add to the script as long as the messages below were incorporated.

***Child 1*** *passes by walking barefoot.*

**Parent 1** – My child keeps walking barefoot. He often complains of a bellyache, he is not growing well and does not always understand things in school.

**Parent 2** – My child is the same, he has a difficult time with school and I do not know why.

***Research staff member 1*** *calls the parents of children for an information session about our research. Several parents are sitting in the room.*

**Research staff member 1** – As you know, our team is here to do research regarding a type of worm called hookworm. My colleague already explained to you our study (*referring to the oral information session these caregivers have already attended*), so would you like to sign or not?

**Parent 3 –** Yes, I would like my daughter to participate in your research.

**Research staff member 1 –** Ok, and how old is she?

**Parent 3 –** She is 2 years old.

**Research staff member 1 –** That will not be possible because we are recruiting children who are 3 to 12 years old only.

**Parent 3 –** Ok, thank you.

**Research staff member 1** – *Looking at parent 2*: And you? Would you like to participate?

**Parent 2** – No! You will tell everyone in our village that my child has worms.

**Research staff member 1** – No, we will not tell anyone any information about your child. Only the researchers and you, the parent, will know his information. We will not even the teacher or your neighbors.

**Parent 2** – Nevertheless, I do not want to participate because I do not believe in those medicines. *He walks away.*

**Parent 1** – Can I sign for his child?

**Research staff member 1** – No, only the parent can sign the consent for their children not even the teacher or the doctor, only the parent.

**Parent 1** – Ah ok, but I have one issue. I want to sign but when I go home and discuss with my partner and he refuses, what will happen?

**Research staff member 1** – There is no problem, you just tell us and your child can still receive treatment if he has worms and you agree.

***Parent 1*** *signs the informed consent form,* ***parent 4*** *refuses and both are given 2 USD for the transport reimbursement and leave.*

**Research staff member 1** – *Hands money to* ***parent 4***: This money is for your transport to come to the meeting.

**Research staff member 1 –** *Gives him a stool container*: Tomorrow your child should bring us the stool sample in this container, ok?

**Parent 4** – Ok.

(The next day)

***Child 1*** *brought his stool, receives a bag of cookies and is given another container for the next day to be checked in the laboratory.*

(The next day)

**Child 1** – Here is my stool sample. *Hands it to* ***research staff member 2****.*

*A* ***lab technician*** *who analyzed the stool sample comes in the room.*

**Lab technician** – We found some children infected. Therefore, they should receive treatment.

(Treatment day)

**Research staff member 1 –** *Talking to the research team*: Please listen to me carefully. Remember that we have 400 children who have hookworm and will be included in the clinical trial. 200 children will receive the chewable mebendazole tablet and 200 will receive the tablet to swallow with water. Do not forget to check if girls are pregnant, if they are 10 years old or more they will need to do a pregnancy test.

***Child 1*** *comes in the room where the clinical analysis and treatment will take place.*

**Nurse 1** – How old are you?

**Child 1** – I am 10.

**Nurse** **1** – Because you are a girl you need to give me a urine sample in this container to do a pregnancy test.

**Child 1** – *Hands container with urine to the nurse*. Here it is.

(Some minutes later)

**Nurse 1** – Ok, you can move on to the other nurse.

**Nurse 2** – Come here, I will take a little bit blood from your finger and look to see if you have anemia. *Pricks the finger and takes a drop of blood*. Ok, now you can go to the doctor over there. *Pointing at the doctor’s desk*.

**Doctor** – How do you feel? *Examines the child’s body and asks him some questions) Ok, now go there and take your medicine.*

**Research staff member 2** – Welcome, I will give you your medicine now.

**Child 1** – How much do I have to pay for the medicine?

**Research staff member 2** – Here medicine is free.

**Child 1** – Oh, ok. I am ready then. What medicine are you giving me?

**Research staff member 2** – It is called mebendazole.

**Child 2** – Does it have any side effect?

**Research staff member 2** – You might feel a little headache or a little dizziness but nothing dangerous.

**Child 2** – Ok.

**Research staff member 2** – So you take this and chew it (*pointing at child 1*), and you take this and swallow it with a glass of water (*pointing at child 2*).

**Child 1** – Can I also chew?

**Research staff member 2** – No, you cannot chew because we want to see, between these two different tablets which one kills more worms and which one you children like more. Now please wait in the next room so we can see if you react well to the medicine.

(3 hours later)

**Doctor** – How do you feel?

**Child 1** – I feel good.

**Doctor** – *Checks for any symptoms with a short questionnaire.* Ok, you can go home now and come back tomorrow for another checkup.

(Next day)

**Doctor** – How do you feel?

**Child 1** – I feel good.

**Doctor** – Ok, thank you. You can go home and wait for another 2 or 3 weeks for another checkup.

(2 to 3 weeks later)

**Research staff member 1** – *Gives out containers to some of the children.* The children who I gave stool containers to, please bring us a stool sample again.

**Child 1** – Why?

**Research staff member 1** – We want to see if the drugs have killed all the worms you had.

(Next day)

***Child 1*** *gives research staff member 1 his stool sample.*

**Research staff member 1** – *Receives the container and hands the child another container.* Here you go another empty container, tomorrow you should bring us a stool again.

**Child 1** – Ok.

*A* ***lab technician*** *that analyzed the stool samples comes in the room.*

**Lab technician** – This child still has hookworm, we need to give him albendazole and ivermectin to treat him.
