# Supplementary material for: Evaluation of two communication tools, slideshow and theater, to improve participants’ understanding of a clinical trial in the informed consent procedure on Pemba Island, Tanzania
Source: PLoS Negl Trop Dis. 2021 May 14;15(5):e0009409. doi: 10.1371/journal.pntd.0009409 (PMC8153490; doi:10.1371/journal.pntd.0009409)
Supplement: S1 Table — (DOCX) [file pntd.0009409.s004.docx]

**S1 Table.** Number (%) of caregivers mentioning each of the key messages spontaneously (with sub-questions; on the left) or responding correctly to the true/false statements concerning each key message not mentioned spontaneously (on the right) by caregiver group (C = control, O = oral information session, S = slideshow both pamphlet and T = theatre). The correct answers to the sub-questions and true/false statements are in bold.

| Spontaneous answers and sub-questions | | | | | | | True/false statements | | | | | |
| --- | --- | --- | --- | --- | --- | --- | --- | --- | --- | --- | --- | --- |
|  |  |  | **C** | **O** | **S** | **T** |  |  | **C** | **O** | **S** | **T** |
| Give us stool samples. | | | 76 (87) | 119 (95) | 136 (93) | 134 (99) | Give us only one stool sample | True | 53 (72) | 15 (94) | 36 (95) | 10 (91) |
|  | How many? | 1 | 12 (16) | 0 (0) | 0 (0) | 0 (0) |  |  |  |  |  |  |
|  |  | 2 | 37 (49) | 117 (98) | 121 (99) | 120 (90) |  | **False** | 2 (3) | 1 (6) | 1 (3) | 0 (0) |
|  |  | 3 | 12 (16) | 1 (1) | 0(0) | 2 (2) |  | No answer | 19 (26) | 0 (0) | 1 (3) | 1 (9) |
|  |  | **4** | 0 (0) | 0 (0) | 11 (8) | 9 (7) | Pay for mebendazole | True | 37 (25) | 31 (23) | 31 (18) | 36 (25) |
|  |  | **2 before and 2 after treatment** | 0 (0) | 0 (0) | 4 (3) | 1 (1) |  | **False** | 88 (59) | 101 (75) | 140 (81) | 107 (74) |
|  |  | Does not know. | 15 (20) | 1 (1) | 0 (0) | 2 (2) |  | No answer | 25 (17) | 3 (2) | 2 (1) | 2 (1) |
| Pay for treatment. | | | 0 (0) | 0 (0) | 1 (1) | 0 (0) | Give us a urine sample for a pregnancy test if she is 9 years old | True | 101 (70) | 111 (93) | 138 (93) | 105 (88) |
| Make a pregnancy test, if a girl. | | | 5 (6) | 15 (12) | 25 (17) | 25 (18) |  | **False** | 17 (12) | 7 (6) | 7 (5) | 11 (9) |
|  | From what age? (years) | 3 | 1 (20) | 0 (0) | 0 (0) | 0 (0) |  | No answer | 27 (19) | 2 (2) | 4 (3) | 4 (3) |
|  |  | 9 | 1 (20) | 2 (13) | 6 (24) | 3 (12) | Not drink water during the day of treatment | True | 36 (24) | 66 (49) | 70 (41) | 60 (41) |
|  |  | **10** | 2 (40) | 10 (67) | 18 (72) | 18 (72) |  | **False** | 83 (56) | 60 (44) | 96 (56) | 78 (54) |
|  |  | 12 | 0 (0) | 0 (0) | 1 (4) | 3 (12) |  | No answer | 30 (20) | 9 (7) | 7 (4) | 7 (5) |
|  |  | Does not know. | 1 (20) | 3 (20) | 0 (0) | 1 (4) | Give us a small blood sample | **True** | 107 (75) | 108 (98) | 127 (96) | 107 (100) |
| Drink water during treatment day. | | | 0 (0) | 0 (0) | 1 (1) | 0 (0) |  | False | 7 (5) | 0 (0) | 3 (2) | 0 (0) |
| Provide a blood sample. | | | 7 (8) | 25 (20) | 42 (29) | 38 (28) |  | No answer | 29 (20) | 2 (2) | 2 (2) | 0 (0) |
|  | A big or a small sample? | **Small** | 6 (86) | 25 (100) | 42 (100) | 38 (100) | Accept that a doctor checks his/her health | **True** | 129 (89) | 124 (98) | 148 (99) | 132 (99) |
|  |  | Does not know. | 1 (14) | 0 (0) | 0 (0) | 0 (0) |  | False | 3 (2) | 0 (0) | 0 (0) | 0 (0) |
| Allow a doctor/nurse to examine him/her. | | | 5 (6) | 9 (7) | 25 (17) | 12 (9) |  | No answer | 13 (9) | 2 (2) | 1 (1) | 1 (1) |
| Provide a urine sample | | | 1 (1) | 0 (0) | 0 (0) | 1 (1) | Tell us if he is feeling well after the medication? | **True** | 109 (73) | 121 (90) | 154 (91) | 133 (92) |
| Collaborate with the study team. | | | 5 (45) | 0 (0) | 0 (0) | 0 (0) |  | False | 5 (3) | 4 (3) | 8 (5) | 2 (1) |
| Unclear answer. | | | 2 (18) | 1 (33) | 3 (100) | 1 (33) |  | No answer | 35 (24) | 9 (7) | 7 /4) | 9 (6) |
| No spontaneous answer (probed for all). | | | 63 (42) | 10 (7) | 27 (16) | 9 (6) | - | - | - | - | - | - |
