# Supplementary material for: Evaluation of two communication tools, slideshow and theater, to improve participants’ understanding of a clinical trial in the informed consent procedure on Pemba Island, Tanzania
Source: PLoS Negl Trop Dis. 2021 May 14;15(5):e0009409. doi: 10.1371/journal.pntd.0009409 (PMC8153490; doi:10.1371/journal.pntd.0009409)
Supplement: S2 Table — (DOCX) [file pntd.0009409.s005.docx]

**S2 Table.** Number (%, using the total number of caregivers giving a spontaneous response as a denominator) of caregiver responses to each question stratified by spontaneous or probed answers and by caregiver group (C = control, O = oral information session, S = slideshow both pamphlet and T = theatre). **Note:** Interviewers recorded all spontaneous answers and caregiver who did not have a spontaneous response where probed using multiple choice (i.e. were given four options and asked to choose one; caregivers who were not able to choose one option were classified as “still does not know”). The answers we considered correct are indicated with an arrow on the left (→). Caregivers often responded more than one spontaneous answer. * questions where there was more than one answer considered correct and, therefore, we present the overlap of both correct answers as well.

|  |  | | | | | Answers | | | | | | | |
| --- | --- | --- | --- | --- | --- | --- | --- | --- | --- | --- | --- | --- | --- |
|  |  | | | | | **Spontaneous** | | | | **Probed** | | | |
| Questions | | | | | | **C** | **O** | **S** | **T** | **C** | **O** | **S** | **T** |
| 1. What is hookworm and how does it infect us? | | | | | **n =** | **87** | **125** | **153** | **137** | **63** | **10** | **21** | **8** |
|  | A worm that can infect us if we drink dirty water. | | | | | 20 (23) | 8 (6) | 5 (3) | 3 (2) | 15 (24) | 1 (10) | 1 (5) | 1 (13) |
| → | A worm that can go into our feet if we walk barefoot. | | | | | 27 (31) | 117 (94) | 146 (95) | 130 (95) | 17 (27) | 7 (70) | 11 (52) | 4 (50) |
|  | A worm that can infect us if we eat rotten food. | | | | | 21 (24) | 5 (4) | 8 (5) | 4 (3) | 10 (16) | 0 (0) | 0 (0) | 1 (13) |
|  | A worm that can get to our food through flies. | | | | | 10 (12) | 1 (1) | 3 (2) | 4 (3) | 11 (18) | 0 (0) | 2 (10) | 0 (0) |
|  | We can become infected if we eat coconut. | | | | | 20 (23) | 2 (2) | 14 (9) | 16 (12) | - | - | - | - |
|  | We can become infected if we eat unwashed fruits. | | | | | 11 (14) | 3 (2) | 5 (3) | 1 (1) | - | - | - | - |
|  | We can become infected if we eat dirty food. | | | | | 6 (7) | 5 (4) | 1 (1) | 3 (2) | - | - | - | - |
|  | We can become infected if we do not eat healthy. | | | | | 4 (5) | 0 (0) | 1 (1) | 4 (3) | - | - | - | - |
|  | We can become infected if we swim in the river. | | | | | 2 (2) | 0 (0) | 0 (0) | 1 (1) | - | - | - | - |
|  | We can become infected if we eat soil. | | | | | 7 () | 0 (0) | 0 (0) | 1 (1) | - | - | - | - |
|  | We can become infected if we eat young mangoes. | | | | | 1 (1) | 0 (0) | 1 (1) | 0 (0) | - | - | - | - |
|  | We can become infected if we eat raw food. | | | | | 3 (3) | 0 (0) | 0 (0) | 0 (0) | - | - | - | - |
|  | We can become infected if we intense oranges. | | | | | 0 (0) | 1 (1) | 0 (0) | 0 (0) | - | - | - | - |
|  | We can become infected if we eat too much. | | | | | 1 (1) | 0 (0) | 0 (0) | 0 (0) | - | - | - | - |
|  | We can become infected if we eat without washing our hands. | | | | | 1 (1) | 0 (0) | 0 (0) | 0 (0) | - | - | - | - |
|  | We can become infected if we play in the dirt/soil. | | | | | 5 (6) | 3 (2) | 1 (1) | 4 (3) | - | - | - | - |
|  | *Unclear answer.* | | | | | 1 (1) | 0 (0) | 0 (0) | 1 (1) | - | - | - | - |
|  | *Still does not know.* | | | | | - | - | - | - | 10 (16) | 2 (20) | 7 (33) | 2 (25) |
| 1. Why is hookworm bad for your child? | | | | | | **119** | **127** | **148** | **132** | **31** | **8** | **26** | **13** |
|  | My child will get pimples all over the body. | | | | | 21 (18) | 14 (11) | 16 (11) | 18 (14) | 4 (13) | 1 (13) | 2 (8) | 1 (8) |
|  | My child’s urine will become red (blood). | | | | | 2 (2) | 0 (0) | 1 (1) | 0 (0) | 2 (7) | 1 (13) | 0 (0) | 1 (8) |
| → | My child may not grow well. | | | | | 49 (41) | 91 (72) | 97 (66) | 96 (73) | 6 (19) | 0 (0) | 17 (65) | 5 (39) |
| → | My child may have difficulties at school. | | | | | 4 (3) | 24 (19) | 48 (32) | 33 (25) |  |  |  |  |
|  | My child will be very hungry all the time. | | | | | 39 (33) | 35 (28) | 36 (24) | 31 (24) | 13 (42) | 4 (50) | 4 (15) | 4 (31) |
|  | My child will not eat. | | | | | 16 (13) | 7 (6) | 5 (3) | 11 (8) | - | - | - | - |
|  | It sucks blood. | | | | | 7 (6) | 2 (2) | 1 (1) | 2 (2) | - | - | - | - |
|  | My child will get a big belly. | | | | | 8 (7) | 4 (3) | 4 (3) | 2 (2) | - | - | - | - |
|  | My child will be tired. | | | | | 10 (8) | 5 (4) | 3 (2) | 6 (5) | - | - | - | - |
|  | My child will get a cough. | | | | | 3 (3) | 0 (0) | 0 (0) | 3 (2) | - | - | - | - |
|  | My child will have shortness of breath. | | | | | 0 (0) | 1 (1) | 0 (0) | 0 (0) | - | - | - | - |
|  | My child will be sick. | | | | | 13 (11) | 6 (5) | 8 (5) | 6 (5) | - | - | - | - |
|  | My child will have a bellyache. | | | | | 7 (6) | 6 (5) | 1 (1) | 5 (4) | - | - | - | - |
|  | My child will have lack of blood (anemia). | | | | | 6 (5) | 2 (2) | 5 (3) | 2 (2) | - | - | - | - |
|  | My child will feel nauseous. | | | | | 2 (2) | 3 (2) | 0 (0) | 0 (0) | - | - | - | - |
|  | My child will vomit. | | | | | 5 (4) | 3 (2) | 1 (1) | 3 (2) | - | - | - | - |
|  | My child will have diarrhea. | | | | | 2 (2) | 0 (0) | 2 (1) | 1 (1) | - | - | - | - |
|  | My child anus will itch. | | | | | 2 (2) | 0 (0) | 0 (0) | 1 (1) | - | - | - | - |
|  | My child will have a fever. | | | | | 2 (2) | 2 (2) | 0 (0) | 1 (1) | - | - | - | - |
|  | My child will have a headache. | | | | | 0 (0) | 4 (3) | 0 (0) | 0 (0) | - | - | - | - |
|  | My child will not see well. | | | | | 1 (1) | 0 (0) | 0 (0) | 0 (0) | - | - | - | - |
|  | My child will feel cold. | | | | | 0 (0) | 0 (0) | 0 (0) | 1 (1) | - | - | - | - |
|  | My child will die. | | | | | 0 (0) | 0 (0) | 1 (1) | 0 (0) | - | - | - | - |
|  | *Unclear answer.* | | | | | 0 (0) | 0 (0) | 0 (0) | 1 (1) | - | - | - | - |
|  | *Still does not know.* | | | | | - | - | - | - | 6 (19) | 2 (25) | 3 (12) | 2 (15) |
| 1. Is it possible to treat hookworm? How? | | | | | | **144** | **133** | **167** | **143** | **6** | **2** | **7** | **2** |
|  | Yes | | | | | 142 (99) | 133 (100) | 166 (99) | 142 (99) | - | - | - | - |
|  |  | It is possible to treat if he/she eats a lot of healthy food like vegetables and fruit. | | | | 0 (0) | 1 (1) | 1 (1) | 0 (0) | 1 (17) | 0 (0) | 0 (0) | 0 (0) |
|  |  | I should take him/her to the traditional healer. | | | | 0 (0) | 1 (1) | 0 (0) | 0 (0) | - | - | - | - |
| → |  | He/she can receive medication that will kill the hookworm. | | | | 139 (95) | 130 (98) | 162 (95) | 140 (97) | 3 (50) | 2 (100) | 5 (71) | 2 (100) |
|  |  | If he/she sees a doctor. | | | | 2 (1) | 1 (1) | 4 (2) | 4 (3) | - | - | - | - |
|  |  | If he/she has good hygiene behavior. | | | | 3 (2) | 1 (1) | 2 (1) | 0 (0) | - | - | - | - |
|  | No | | | | | 2 (1) | 0 (0) | 1 (1) | 1 (1) | 1 (17) | 0 (0) | 1 (14) | 0 (0) |
|  | *Still does not know.* | | | | | - | - | - | - | 1 (17) | 0 (0) | 1 (14) | 0 (0) |
| 1. Why are we doing this study and what do we want to find out? * | | | | | | **24** | **88** | **112** | **100** | **126** | **47** | **62** | **45** |
|  | To see if mebendazole kills the worms in my child’s belly because this drug has never been used before. | | | | | 4 (17) | 6 (7) | 6 (5) | 5 (5) | 10 (8) | 7 (15) | 16 (26) | 2 (4) |
|  | To see if mebendazole kills the worms that are in my child’s feet. | | | | | 2 (8) | 16 (18) | 19 (17) | 6 (6) | 27 (21) | 5 (11) | 17 (27) | 8 (18) |
|  | We want to see if mebendazole is better than another drug called ivermectin at killing the worms. | | | | | 1 (4) | 16 (18) | 13 (12) | 19 (19) | 13 (10) | 6 (13) | 2 (3) | 5 (11) |
| → | We want to find out if the new chewable mebendazole  kills more hookworm than the old solid type. | | | | | 3 (13) | 25 (28) | 45 (40) | 30 (30) | 23 (18) | 18 (38) | 17 (27) | 20 (44) |
| → | We want to see if children like better the chewable or the solid tablet. | | | | | 2 (8) | 5 (6) | 3 (3) | 11 (11) | - | - | - | - |
| → | The two answers above. | | | | | 0 (0) | 0 (0) | 0 (0) | 0 (0) | - | - | - | - |
|  | We want to treat children. | | | | | 12 (50) | 20 (23) | 22 (17) | 31 (31) | - | - | - | - |
|  | We want to educate children. | | | | | 0 (0) | 0 (0) | 1 (1) | 1 (1) | - | - | - | - |
|  | We are doing research. | | | | | 0 (0) | 0 (0) | 3 (3) | 0 (0) | - | - | - | - |
|  | We want to check if children have worms. | | | | | 1 (4) | 3 (3) | 3 (3) | 6 (6) | - | - | - | - |
|  | *Unclear answer.* | | | | | 0 (0) | 2 (2) | 0 (0) | 0 (0) | - | - | - | - |
|  | *Still does not know.* | | | | | - | - | - | - | 53 (42) | 11 (23) | 10 (16) | 10 (22) |
| 1. Who should decide if your child should participate in this study? | | | | | | **149** | **135** | **173** | **145** | **1** | **0** | **1** | **0** |
|  | The teacher. | | | | | 3 (2) | 2 (2) | 4 (2) | 7 (5) | 0 (0) | 0 (0) | 0 (0) | 0 (0) |
| → | The parents (me) | | | | | 147 (99) | 134 (99) | 172 (99) | 143 (99) | 0 (0) | 0 (0) | 1(100) | 0 (0) |
|  | The doctor. | | | | | 3 (2) | 5 (4) | 6 (3) | 2 (1) | 0 (0) | 0 (0) | 0 (0) | 0 (0) |
|  | The nurse | | | | | 0 (0) | 0 (0) | 1 (1) | 0 (0) |  |  |  |  |
|  | A neighbor. | | | | | 0 (0) | 0 (0) | 0 (0) | 0 (0) | 0 (0) | 0 (0) | 0 (0) | 0 (0) |
|  | A friend | | | | | 0 (0) | 0 (0) | 1 (1) | 0 (0) |  |  |  |  |
|  | The child him/herself. | | | | | 0 (0) | 1 (1) | 0 (0) | 0 (0) | - | - | - | - |
|  | *Still does not know.* | | | | | - | - | - | - | 1 (100) | 0 (0) | 0 (0) | 0 (0) |
| 1. What happens if your child still has worms after the treatment? | | | | | | **127** | **132** | **157** | **142** | **23** | **3** | **17** | **3** |
|  | There is nothing we can do. | | | | | 4 (3) | 5 (4) | 0 (0) | 1 (1) | 1 (4) | 0 (0) | 0 (0) | 0 (0) |
|  | We will give him/her an injection to kill the worms. | | | | | 1 (0) | 0 (0) | 0 (0) | 0 (0) | 0 (0) | 0 (0) | 0 (0) | 0 (0) |
|  | My child should drink a lot of water. | | | | | 0 (0) | 0 (0) | 0 (0) | 1 (1) | 0 (0) | 0 (0) | 0 (0) | 0 (0) |
| → | We will give him/her another medicine to kill the worms. | | | | | 116 (91) | 124 (94) | 153 (98) | 136 (96) | 15 (65) | 3 (100) | 16 (94) | 3 (300) |
|  | We will treat him/her again. | | | | | 4 (3) | 1 (1) | 4 (3) | 5 (4) | - | - | - | - |
|  | I will find another solution. | | | | | 1 (1) | 0 (0) | 0 (0) | 0 (0) | - | - | - | - |
|  | We will educate him about the impact of these worms. | | | | | 1 (1) | 0 (0) | 0 (0) | 0 (0) | - | - | - | - |
|  | I will ask the doctor for advice. | | | | | 4 (3) | 1 (1) | 4 (3) | 6 (4) | - | - | - | - |
|  | *Unclear answer.* | | | | | 1 (1) | 3 (2) | 0 (0) | 1 (1) | - | - | - | - |
|  | *Still does not know.* | | | | | - | - | - | - | 7 (30) | 0 (0) | 1 (6) | 0 (0) |
| 1. Can your child give up participating during the study? | | | | | | **128** | **122** | **166** | **139** | **22** | **13** | **8** | **6** |
|  | Yes | | | | | 17 (13) | 34 (28) | 29 (17) | 32 (23) | - | - | - | - |
| → |  | And there is no consequence, he/she will still receive treatment. | | | | 7 (41) | 9 (26) | 13 (45) | 11 (34) | 0 (0) | 0 (0) | 1 (13) | 0 (0) |
|  |  | But he/she will not receive treatment. | | | | 7 (41) | 23 (68) | 15 (52) | 19 (59) | 1 (5) | 1 (8) | 2 (25) | 1 (17) |
|  |  | And we will have to discuss what to do. | | | | 1 (6) | 0 (0) | 0 (0) | 0 (0) | - | - | - | - |
|  |  | But he/she will stay infected. | | | | 2 (12) | 2 (6) | 0 (0) | 1 (3) | - | - | - | - |
|  |  | *But does not know if the child will still be treated or not.* | | | | 2 (12) | 3 (9) | 3 (10) | 1 (3) | - | - | - | - |
|  | No | | | | | 111 (87) | 88 (72) | 137 (83) | 107 (77) | - | - | - | - |
|  |  | No, he/she cannot give up if I decide he participates he has to stay until the end of the study. | | | | 79 (71) | 69 (78) | 103 (75) | 71 (92) | 5 (23) | 3 (23) | 0 (0) | 2 (33) |
|  |  | Only if the doctor and teacher agree that he can give up. | | | | 6 (5) | 1 (1) | 7 (5) | 9 (12) | 7 (32) | 7 (54) | 4 (50) | 1 (17) |
|  |  | No but does not know why (moved to probed). | | | | 9 (8) | 6 (7) | 7 (5) | 8 (10) | - | - | - | - |
|  |  | No, I do not allow him to give up. | | | | 0 (0) | 0 (0) | 1 (1) | 1 (1) | - | - | - | - |
|  |  | No, because he/she is not yet cured. | | | | 20 (18) | 12 (14) | 18 (13) | 20 (26) | - | - | - | - |
|  |  | No, he/she does not decide. | | | | 1 (1) | 2 (2) | 1 (1) | 0 (0) | - | - | - | - |
|  |  | No, because of the research. | | | | 0 (0) | 0 (0) | 1 (1) | 1 (1) | - | - | - | - |
|  |  | No, because he/she already accepted. | | | | 0 (0) | 1 (1) | 0 (0) | 0 (0) | - | - | - | - |
|  | *Unclear answer.* | | | | | 1 (1) | 0 (0) | 1 (1) | 0 (0) | - | - | - | - |
|  | *Still does not know.* | | | | | - | - | - | - | 9 (41) | 2 (15) | 1 (13) | 2 (33) |
| 1. What about payment? Do you pay us? Do we pay you? How does it work? | | | | | | **110** | **130** | **163** | **143** | **40** | **5** | **11** | **2** |
|  | There are some costs for you: you will have to pay for your child’s treatment. | | | | | 16 (15) | 2 (2) | 3 (2) | 14 (10) | 3 (8) | 0 (0) | 0 (0) | 0 (0) |
|  | You don’t pay for anything | | | | | 21 (19) | 63 (49) | 71 (44) | 59 (41) | 15 (38) | 5 (100) | 10 (91) | 2 (100) |
|  | You will receive 2$ | | | | | 7 (6) | 45 (35) | 72 (44) | 58 (41) |  |  |  |  |
|  | We will pay you | | | | | 65 (59) | 27 (21) | 23 (14) | 29 (20) |  |  |  |  |
|  | You will receive money if your child accepts the treatment | | | | | - | - | - | - | 1 (3) | 0 (0) | 0 (0) | 0 (0) |
|  | You will only get money if the treatment kills the worms | | | | | 0 (0) | 0 (0) | 0 (0) | 0 (0) | 4 (10) | 0 (0) | 0 (0) | 0 (0) |
|  | I do not have money. | | | | | 0 (0) | 0 (0) | 0 (0) | 0 (0) | - | - | - | - |
|  | As the study team prefers. | | | | | 1 (1) | 0 (0) | 0 (0) | 0 (0) | - | - | - | - |
|  | *Unclear answer.* | | | | | 0 (0) | 0 (0) | 1 (1) | 0 (0) | 0 (0) | - | - | - |
|  | *Still does not know.* | | | | | - | - | - | - | 17 (43) | 0 (0) | 1 (9) | 0 (0) |
| 1. Is mebendazole safe for your child? Will he/she feel something? | | | | | | **92** | **122** | **157** | **135** | **58** | **13** | **17** | **10** |
|  | Yes | | | | | 89 (97) | 120 (98) | 157 (100) | 130 (96) | - | - | - | - |
|  |  | I do not know if he/she will feel something or not. | | | | 11 (12) | 5 (4) | 6 (4) | 4 (3) | - | - | - | - |
|  |  | He/she will not feel anything at all. | | | | 23 (26) | 38 (32) | 47 (30) | 43 (33) | 15 (26) | 8 (62) | 8 (47) | 7 (70) |
| → |  | But he/she may feel some side effects. | | | | 55 (62) | 77 (63) | 104 (66) | 83 (64) | 3 (5) | 1 (8) | 2 (12) | 0 (0) |
|  |  |  | What side effects may he/she feel? * | | |  |  |  |  |  |  |  |  |
|  |  |  | → | Dizziness | | 22 (40) | 52 (68) | 81 (78) | 59 (71) | - | - | - | - |
|  |  |  | → | Bellyache | | 15 (27) | 34 (44) | 43 (41) | 36 (43) | - | - | - | - |
|  |  |  |  | Headache | | 10 (18) | 32 (42) | 45 (43) | 40 (48) | - | - | - | - |
|  |  |  |  | Nausea | | 4 (73) | 4 (5) | 2 (2) | 4 (5) | - | - | - | - |
|  |  |  |  | Tired | | 5 (9) | 0 (0) | 0 (0) | 0 (0) | - | - | - | - |
|  |  |  |  | Diarrhea | | 2 (4) | 0 (0) | 1 (1) | 0 (0) | - | - | - | - |
|  |  |  |  | Vomiting | | 1 (2) | 0 (0) | 8 (8) | 3 (4) | - | - | - | - |
|  |  |  |  | Fever | | 1 (2) | 0 (0) | 0 (0) | 2 (2) | - | - | - | - |
|  |  |  |  | Sleepy | | 0 (0) | 0 (0) | 0 (0) | 1 (1) | - | - | - | - |
|  |  |  |  | Insomnia | | 0 (0) | 0 (0) | 1 (1) | 0 (0) | - | - | - | - |
|  |  |  |  | The worms will come out of him/her. | | 1 (2) | 0 (0) | 0 (0) | 1 (1) | - | - | - | - |
|  |  |  |  | He/she will be healthy again. | | 2 (4) | 2 (3) | 4 (4) | 0 (0) | - | - | - | - |
|  |  |  |  | It has already been tested. | | 0 (0) | 2 (3) | 1 (1) | 1 (1) | - | - | - | - |
|  |  |  |  | It is still being studied. | | 1 (2) | 0 (0) | 1 (1) | 0 (0) | - | - | - | - |
|  |  |  |  | Only God knows. | | 1 (2) | 0 (0) | 0 (0) | 0 (0) | - | - | - | - |
|  |  |  |  | *Does not know what he/she will feel.* | | 3 (5) | 3 (4) | 0 (0) | 1 (1) | - | - | - | - |
|  | No | | | | | 3 (3) | 2 (2) | 0 (0) | 5 (4) | - | - | - | - |
|  |  | What will he/she feel? | | | |  |  |  |  |  |  |  |  |
|  |  |  | If your child takes the treatment he will not be able to walk for a few days. | | | 0 (0) | 0 (0) | 0 (0) | 3 (60) | 2 (3) | 0 (0) | 0 (0) | 0 (0) |
|  |  |  | If your child takes the treatment he will sleep all day. | | | 1 (33) | 1 (50) | 0 (0) | 2 (40) | - | - | - | - |
|  |  |  | He/she will get drunk. | | | 1 (33) | 0 (0) | 0 (0) | 0 (0) | - | - | - | - |
|  |  |  | *Does not know what he/she will feel.* | | | 1 (33) | 1 (50) | 0 (0) | 0 (0) | - | - | - | - |
|  | *Still does not know.* | | | | | - | - | - | - | 38 (66) | 4 (31) | 7 (41) | 3 (30) |
| 1. Who will be able to see your child’s personal information? * | | | | | | **143** | **134** | **173** | **145** | **7** | **1** | **1** | **0** |
|  | Neighbors | | | | | 0 (0) | 1 (1) | 0 (0) | 0 (0) | 1 (14) | 0 (0) | 1 (100) | 0 (0) |
|  | Parent | | | | | 2 (1) | 3 (2) | 1 (1) | 1 (1) |  |  |  |  |
|  | Study investigators | | | | | 24 (17) | 21 (16) | 31 (18) | 26 (18) | - | - | - | - |
| → | Parents and study investigators only | | | | | 21 (15) | 32 (24) | 45 (26) | 52 (36) | 1 (14) | 1 (100) | 0 (0) | 0 (0) |
|  | Only parents | | | | | 94 (66) | 78 (58) | 95 (55) | 66 (46) | 1 (14) | 0 (0) | 0 (0) | 0 (0) |
|  | Teacher | | | | | 4 (3) | 2 (2) | 2 (1) | 3 (2) | - | - | - | - |
|  | Doctor | | | | | 0 (0) | 0 (0) | 1 (1) | 0 (0) | - | - | - | - |
|  | *Unclear answer.* | | | | | 0 (0) | 0 (0) | 1 (1) | 0 (0) | - | - | - | - |
|  | *Still does not know.* | | | | | - | - | - | - | 4 (57) | 0 (0) | 0 (0) | 0 (0) |
